# Evasion of host antiviral innate immunity by HSV-1, an update

**DOI:** 10.1186/s12985-016-0495-5

**Published:** 2016-03-08

**Authors:** Chenhe Su, Guoqing Zhan, Chunfu Zheng

**Affiliations:** Institutes of Biology and Medical Sciences, Soochow University, Suzhou, 215123 China; Department of Infectious Disease, Renmin Hospital, Hubei University of Medicine, Shiyan, Hubei 442000 China; Department of Microbiology, Immunology and Infectious Deseases, University of Calgary, Calgary, AB T2N 4N1 Canada

**Keywords:** HSV-1, IFN, Antiviral innate immunity, Immune evasion

## Abstract

Herpes simplex virus type 1 (HSV-1) infection triggers a rapid induction of host innate immune responses. The type I interferon (IFN) signal pathway is a central aspect of host defense which induces a wide range of antiviral proteins to control infection of incoming pathogens. In some cases, viral invasion also induces DNA damage response, autophagy, endoplasmic reticulum stress, cytoplasmic stress granules and other innate immune responses, which in turn affect viral infection. However, HSV-1 has evolved multiple strategies to evade host innate responses and facilitate its infection. In this review, we summarize the most recent findings on the molecular mechanisms utilized by HSV-1 to counteract host antiviral innate immune responses with specific focus on the type I IFN signal pathway.

## Background

Herpes simplex virus type 1 (HSV-1) is a typical human-restricted pathogen which is carried by 50–90 % of the population worldwide, with the higher frequencies in developing countries. HSV-1 is well known for its ability to establish a lifelong latent infection in neurons and trigger reactivation and lytic infection mainly in epithelial or mucosal cells [1–3].

The type I interferon (IFN) signal pathway is the crucial first line of defense and mediates a wide range of innate immune responses toward viral infection. The type I IFN signal pathway is activated upon recognition of viral constituents by pattern-recognition receptors (PRRs), then exerts its function through the expression of multiple IFN-stimulated genes (ISGs) [4, 5]. Cytosolic PRRs include several members of the Toll-like receptor (TLRs) family and certain DNA and RNA sensors [6]. TLRs are the first to be discovered and identified PRRs that detect pathogen-associated molecular patterns. TLR3, TLR7, TLR8, and TLR9 locate on the endosomal membrane and detect nucleic acids [7, 8]. RIG-I-like receptors (RLRs), including retinoic acid-inducible gene I (RIG-I) and melanoma differentiation-associated gene 5 (MDA5) and other RNA receptors can detect distinct RNA structures, while cytoplasmic DNA is detected by recently discovered DNA sensors, including cyclic GMP-AMP synthase (cGAS), IFN-γ-inducible protein 16 (IFI16), DEAD box polypeptide 41 (DDX41), DNA-dependent activator of IRFs (DAI) and several proteins involved in the DNA damage response [6, 9–11].

Apart from the type I IFN signal pathway, the host has also evolved other host antiviral innate immune responses to counteract viral infection. Chromosomal breaks at specific sites were induced upon HSV-1 infection, and viruses interact with cellular pathways responsible for recognition and repair of DNA lesions also known as the DNA damage response (DDR). Recent studies show that certain aspects of DDR play a positive role on antiviral defense [12, 13]. Autophagy, a conserved cell-autonomous pathway, is involved in various physiological processes, including antiviral responses [14]. The canonical function of autophagy allows the regular degradation and recycling of cellular components through isolating targeted cytoplasmic constituents within a double-membraned vesicle known as an autophagosome and exerts several different antiviral roles. Previous study indicates that HSV-1 infection leads to endoplasmic reticulum (ER) stress, which could restrict viral pathogenesis through modulating the immune responses or causing apoptosis [15]. Moreover, viral infection also induces the formation of cytoplasmic granules known as stress granules (SGs). Evidence shows that there is a strong correlation among SG formation, type I IFN production and viral propagation, which suggests that SGs could induce innate responses and restrain viral infection [16].

However, to evade host innate responses, HSV-1 has developed multiple mechanisms to attenuate host antiviral elements and facilitate its infection. In this review, we will discuss the most recent findings on the molecular mechanisms utilized by HSV-1 to evade host antiviral innate immune responses. Specific attention will be given to type I IFN signal pathway, as it plays a central role in the innate antiviral immunity.

## HSV-1 infection dampens the type I interferon production and its downstream signaling pathway

### TLR signaling pathway

TLRs play a pivotal role in host defense against HSV-1 by detecting pathogen-associated molecular patterns and subsequently recruiting downstream adaptor proteins such as myeloid differentiation primary response protein 88 (MyD88), MyD88 adaptor-like protein (Mal), Toll/interleukin 1 receptor domain-containing adaptor protein (TIRAP), Toll/interleukin 1 receptor domain-containing adaptor-inducing IFN-β (TRIF), and/or TRIF-related adaptor molecule (TRAM). This leads to the formation of distinct protein complexes, including TANK-binding kinase 1 (TBK1) and inducible inhibitor of κB kinase (IKK), which then activate transcription factors interferon regulatory factor 3 (IRF3) and interferon regulatory factor 7 (IRF7) to further induce the expression of IFN-β. Until now, TLR2, TLR3, TLR4 and TLR9 are reported to recognize HSV-1 elements [8, 17, 18].

#### TLR3

TLR3 is well characterized for its ability to induce the expression of type I IFNs and inflammatory cytokines following detection of double-strand RNA, and is the only TLR reported to play a non-redundant role in host antiviral immunity. TLR3 deficient fibroblasts produced much less IFN-I upon HSV-1 infection compared to controls, and impaired TLR3 signaling also results in high levels of viral replication [19–21]. Peri et al. reported that HSV-1 tegument protein kinase US3 could reduce the expression of TLR3 thus inhibiting TLR3-mediated response, and depletion of US3 resulted in strong activation of IRF3 and the type I IFN response [22].

#### MyD88

MyD88, composed of a Toll-interleukin-1 receptor domain and a death domain, is a crucial adapter molecule in the TLR signal pathway [23]. Van Lint et al. reported that expression of ICP0 alone was sufficient to reduce the levels of MyD88 and Mal (TIRAP) through its E3 ligase function and cellular proteasomal activity, thus reduced the TLR2-mediated inflammatory response against HSV-1 infection [24].

#### TRAF6

TNF receptor associated factor 6 (TRAF6) is a member of TRAF proteins, which play an important role as adapter molecules to transmit intracellular signals [25]. TRAF6 interacts with TAK1 and the IKK complex, leading to activation of the IKK complex, the latter then phosphorylates the inhibitor of κB, causing its ubiquitination and degradation [26]. HSV-1 kinase US3 could also inhibit TLR2 signaling by reducing TRAF6 polyubiquitination which depends on its kinase activity [27].

### RLR signaling pathway

#### RIG-I and MAD5

RLRs, best represented by RIG-I and MDA5, are proven to play a crucial role during HSV-1 infection. RIG-I mainly recognizes RNA bearing 5’-triphosphate group, while MDA5 typically recognizes dsRNA that is over 2000 bps in length. Both RIG-I and MDA5 could transmit signals to the downstream adaptor protein mitochondrial antiviral-signaling protein (MAVS also known as IPS-1, VISA, or CARDIF), which subsequently activates IRF3 and NF-κB and triggers the expression of type I IFNs and inflammatory cytokines [28–30]. Previous studies in our lab have demonstrated that several HSV-1 proteins could down-regulate the RLR signaling pathway via targeting multiple proteins involved in this pathway with distinct mechanisms. US11, an RNA binding tegument protein of HSV-1 [31], interacts with endogenous RIG-I and MDA5 via its carboxyl-terminal amino acids in an RNA independent manner, and subsequently impedes the formation of RIG-I/MAVS and MDA5/MAVS complexes, resulting in reduced production of IFN-β [32].

#### TRAF3

TRAF3 is a crucial component of the RLR-mediated signaling pathway and links upstream type I IFN signaling responses of MAVS to TBK1 [33, 34]. K63-linked polyubiquitination of TRAF3 is indispensable for signaling by MAVS and recruiting the kinases TBK1 and IκB kinase ε (IKKε), leading to IRF3 phosphorylation and subsequent type I IFN production. As the largest tegument protein of HSV-1, UL36 is essential for HSV-1 replication and is conserved across the herpesviridae family. UL36 contains a deubiquitinase (DUB) motif within its N terminus also known as UL36 ubiquitin-specific protease (UL36USP) [35, 36]. The DUB activity of UL36USP is crucial to block IFN-β production via deubiquitinating TRAF3 thus preventing the recruitment of the downstream adaptor TBK1 [37].

#### TBK1

TBK1 is an IκB Kinase-related kinase phosphorylating a wide range of substrates involved in several cellular processes. TBK1 is activated upon recognition of nucleic acids by various DNA and RNA sensors, and triggers phosphorylation of IRF3, activation of NF-κB and the expression type I IFN. HSV-1 ICP34.5, a multifunctional neurovirulence factor, contains 263 amino acids and is essential in viral pathogenesis [38]. It has been shown that HSV-1 ICP34.5 could inhibit IFN production by binding and sequestering TBK1 [39, 40].

#### IRF3

IRF3 plays a crucial role in the innate immune responses to viral infection, especially on activating the transcription of type I IFN, and dimerization of IRF3 is a distinguishing feature of early activation of the antiviral responses. HSV-1 US3 is a serine/threonine protein kinase that is also conserved across the herpesviridae family [41]. HSV-1 kinase US3 could significantly down regulate the activation of type I IFN and IFN-stimulated response element (ISRE) depending on its protein kinase activity. Moreover, US3 hyperphosphorylates IRF3 to induce untypical phosphorylation of IRF3 and inhibits its dimerization and nuclear translocation. Infection of US3 KD mutant viruses, K220M and D305A respectively, resulted in a much robust IFN-β production compared with wild-type virus [42]. VP16 is an abundant 65-kDa virion phosphoprotein that is delivered by the infecting virions and functions during the earliest stages of infection to stimulate transcription of the viral immediate-early genes, thereby facilitating the onset of the lytic program of viral gene expression [43]. Our previous research revealed that VP16 could block the transactivation activity of IRF-3 through interaction with IRF3 and interfering IRF-3 to recruit its coactivator CBP [44]. ORF61 protein from varicella-zoster virus is homologous to HSV-1 protein ICP0 [45]. Distinct from that of ICP0, ORF61 inhibits IFN-β production through degrading phosphorylated IRF3 via the ubiquitin-proteasome pathway [46].

#### NF-κB

Activation of most PRRs, for example TLRs and RLRs, leads to the induction of NF-κB signaling, which mediates the expression of a large number of cytokines involved in the innate immune responses, especially in the type I IFN signal pathway [47, 48]. Recently, it is reported that NF-κB also contributes to STING dependent, dsDNA-mediated type I IFN production [49]. The p65/RelA subunit of NF-κB is a crucial transcription factor in host innate immune system. Many viruses, including RNA and DNA viruses, antagonize the host inflammation responses at the p65/RelA level. Conserved within alphaherpesvirus, US3 hyperphosphorylates p65 at serine 75 and abrogates its nuclear translocation, resulting in inhibiting NF-κB activation and decreasing expression of many inflammatory chemokine [50]. HSV-1 ICP0 is an immediate early and multifunctional protein that plays a pivotal role during both lytic and latent infections. ICP0 has an E3 ubiquitin ligase activity that promotes degradation of certain host proteins [51, 52]. Our previous study shows that ICP0 significantly suppresses TNF-α-mediated NF-κB activation through binding to the NF-κB subunits p65 and p50, and ICP0 abolishes nuclear translocation of p65 and degrades p50 through the ubiquitin-proteasome pathway depending on its E3 ligase activity [53].

### DNA sensors signaling pathway

Cytosolic DNA was known to induce immune responses but the underlining mechanisms were not understood until the recent discovery of multiple DNA sensors. Ishii et al. found that double-strand B-form DNA could trigger innate antiviral responses including production of type I IFN and chemokines independent of TLRs or RIG-I [54]. During the past few years, great progress has been made in discovering and characterizing several important cytosolic DNA sensors which has greatly improved our understanding of mechanisms of DNA sensor signaling pathway.

#### STING

Growing evidence shows that stimulator of interferon genes (STING, also known as TMEM173, MITA, ERIS and MPYS), expressed in T cells, DCs, macrophages, endothelial cells, epithelial cells and fibroblasts, is an important adaptor for type I IFN induction by cytosolic DNA and plays a critical role in innate immune signaling [55–58]. STING is anchored in the endoplasmic reticulum (ER) in unstimulated cells through four transmembrane domains residing in its N-terminal region, while its C terminus recruits TBK1 and IRF3, and facilitates the phosphorylation of IRF3 by TBK1. STING was recently identified as an ISG and ectopic expression of STING in HEK293T cells could induce robust production of IFN-β [59]. STING knockout mice were significantly more susceptible to HSV-1 compared with the wild-type siblings [60], suggesting that STING might play an important role in host restriction of HSV-1 infection. However, till now, little is known about how HSV-1 evades STING mediated immune responses. Kalamvoki et al. reported that HSV-1 infection affected the stability and function of STING in a cell dependent manner, which depended on the functional integrity of ICP0 and US3-PK [61].

#### cGAS

cGAS, also known as MB21D1 and C6orf150, belongs to the nucleotidyltransferase family and shares highly related structural and enzymatic features with the well-known dsRNA-sensing 2ʹ–5ʹ-oligoadenylate synthase (OAS) proteins [62]. Using biochemical purification and quantitative mass spectrometry, Sun et al. identified cGAS as a novel and main sensor for cytosolic DNA [63, 64]. cGAS, as well as STING, is an ISG, which undergoes a significant conformational change after dsDNA binding and catalyzes ATP and GTP to produce cyclic GMP-AMP (cGAMP), an endogenous second messenger [63, 65, 66]. The latter then binds to and induces dramatic conformational change of STING, leading to its activation and subsequent signal transduction. Depletion of cGAS using shRNA abolished DNA induced activation of IRF3 and IFN-β production. Similarly, cGAS knockout mice were significantly more susceptible to HSV-1 infection compared with the wild-type siblings, indicating that just as STING, cGAS also played an important role in host defense against HSV-1 infection [67]. Surprisingly, Orzalli et al. reported that cGAS produced low amounts of cGAMP upon HSV-1 infection whereas IFN-γ-inducible protein 16 (IFI16) played a direct role in HSV DNA sensing [68]. Since cGAS mediated signal transduction is completely dependent on STING, viral proteins affecting STING or its downstream molecules may directly affect the cGAS pathway. Nonetheless, none of HSV-1 proteins has been reported to specifically antagonize the cGAS/STING mediated cytosolic DNA sensing signal pathway yet. A high-throughput screen assay of all HSV-1 proteins was performed in our lab to identify viral proteins involving in immune evasion of the cGAS-STING signal pathway.

#### IFI16

IFI16 is a DNA binding protein and belongs to the PYHIN family that contains an N-terminal pyrin domain and two C-terminal HIN domains. Unlike other cellular DNA sensors, IFI16 shuttles between the nucleus and the cytoplasm, and is predominantly nuclear at resting state [69–71]. Upon HSV-1 infection, IFI16 recognizes and directly associates with viral genome in the nucleus, then interacts with histone acetyltransferase p300. Acetylation of IFI16 results in its cytoplasmic redistribution where it interacts with STING, and subsequently induces IRF-3 phosphorylation as well as interferon-β production to counteract viral replication [72]. One study showed that IFI16 bound to HSV-1 promoters and prevented association of transcriptional activators with HSV-1 promoters, thus restricted HSV-1 replication through modulating histone modifications [73]. Johnson et al. and Orzalli et al. reported that HSV-1 specifically targeted IFI16 for proteasomic degradation depending on its E3 ubiquitin protein ligase ICP0 [74, 75], while another report presented that expression of ICP0 alone was not sufficient to degrade IFI16 and infection with ICP0-null HSV-1 could also result in IFI16 degradation [76], suggesting that other viral proteins may also be involved in this process. HSV-1 infection could also induce the activation of IFI16 leading to the formation of the IFI16-ASC (apoptosis-associated speck-like protein containing CARD)-procaspase-1 inflammasome complex and maturation of IL-1β early during infection. ICP0 targeted IFI16 for degradation at later times to evade the proinflammatory consequences [74]. Further studies are necessary to fully understand dynamic interaction between IFI16 and ICP0 and other viral proteins.

#### Other DNA sensors

Several proteins were identified as candidate cytosolic DNA sensors before the discovery of cGAS, such as DAI, DDX41, DNA-dependent protein kinase (DNA-PK) et al. [6, 10]. Though these proteins were reported to play a role on viral restriction, further studies showed that they were dispensable for DNA-induced responses in many human cells, suggesting that they might play a redundant or cell-type specific role.

### Interferon-stimulated genes

Type I IFNs trigger the induction of numerous ISGs, including viperin, zinc finger antiviral protein (ZAP), tetherin, double stranded RNA-dependent protein kinase (PKR), 2’-5’ oligoadenylate synthetase (OAS), etc., and distinct sets of ISGs cooperate to reinforce type I IFN signaling and prime cells with enhanced antiviral activity to inhibit viral replication. Here, we discuss how HSV-1 evades the antiviral activity of the aforementioned ISGs.

#### Viperin

Viperin (also known as RSAD2 or Cig5) is one of the well-studied ISGs highly induced through both type I IFN-dependent and type I IFN-independent pathways, and restricts a broad range of viruses. HSV-1 UL41 was an endoribonuclease with a substrate specificity similar to that of RNase A [77]. Previous studies in our lab showed that wild-type (WT) HSV-1 infection couldn’t induce viperin production, and ectopically expression of viperin inhibited the replication of UL41-null HSV-1 but not WT viruses [78]. The underlying molecular mechanism is that UL41 counteracts viperin’s antiviral activity by reducing its mRNA accumulation [78].

#### ZAP

ZAP is a host restriction factor that prevents the accumulation of viral mRNA in the cytoplasm through interacting with the ZAP-responsive elements in the viral RNA [79]. HSV-1 UL41 protein abrogates the antiviral activity of human ZAP by targeting its mRNA for degradation, and consequently inhibiting the expression of hZAP [80].

#### Tetherin

Tetherin (also known as BST2 and CD317) is an ISG which was initially identified as an antiviral effector by inhibiting enveloped virus release without interaction with viral proteins [81]. Tetherin was recently demonstrated to restrict HSV-1 replication by suppressing virus release, and HSV-1 envelope gM was identified as an antagonist to tetherin restriction [82]. Further investigation showed that HSV-1 efficiently depleted tetherin from infected cells by reducing its mRNA via UL41, and that depletion of tetherin could compensate for defects in replication and release of UL41-null HSV-1 [83].

#### PKR

PKR is activated by dsRNA introduced to cells by many viruses since dsRNA is a frequent by-product of viral replication. Upon activation, PKR subsequently phosphorylates the eukaryotic translation initiation factor EIF2A, which leads to the inhibition of cellular mRNA translation, thereby preventing viral protein synthesis and blocking viral production [84]. A recent report by Low-Calle et al. showed that HSV-1 infection decreased PKR constitutive expression [85].

#### OAS

Similar to PKR, OAS is also a key dsRNA-responsive effector. There are three major forms of OAS, all of which recognize dsRNA through a positively charged groove in the molecule. OAS undergoes a conformational change after binding dsRNA and results in the synthesis of 2’,5’-oligoadenylates (2-5As) [86]. These 2-5As products subsequently activate latent RNase L, resulting in both viral and endogenous RNA degradation and the inhibition of viral replication [87]. Sànchez et al. reported that HSV-1 US11 protein was sufficient to block OAS activation depending on its dsRNA-binding domain, and the underlining mechanism could be that US11 sequestered available dsRNA produced during infection [88].

## Other host antiviral innate immune responses

### DDR

DDR is an evolutionarily conserved response to detect DNA damage and initiate cell-cycle arrest to repair the damage. DDR pathways are mainly controlled by ataxia telangiectasia mutated (ATM), ataxia telangiectasia and Rad3 related (ATR) and DNA-PK. Viral DNA genome can be sensed by DDR components which lead to the inhibition of viral replication [12, 13, 89]. Recent researches showed that several DDR sensors, such as DNA-PK and MRE11, were also involved in cytosolic DNA sensing and subsequently type I IFN production [90, 91]. HSV-1 IE gene product ICP0 was reported to prevent full activation of ATM response by promoting the proteasome mediated degradation of DNA-PK, RNF8 and RNF168 [92, 93]. Parkinson et al. found that ICP0 alone was sufficient to degrade DNA-PKcs, the catalytic subunit of DNA-PK, and virus yield was enhanced following infection of DNA-PK negative cells. Chaurushiya et al. reported a novel mechanism utilized by ICP0 to degrade RNF8 [94]. During HSV-1 infection, ICP0 is phosphorylated by cellular CK1 kinase specifically on T67, which facilitates its interaction with RNF8 via the fork-head associated (FHA) domain and promotes RNF8 degradation [94].

### Autophagy

Autophagy is an ancient cell-autonomous defense mechanism and plays a crucial antiviral role in restricting viral invasion [14]. Most recently, Liang et al. found that the autophagy protein Beclin1 directly interacted with cytosolic DNA sensor cGAS, resulting in the inhibited production of cGAMP by cGAS and enhanced autophagy-mediated degradation of cytosolic DNAs [95]. Surprisingly, autophagy deficiency does not significantly increase the viral infectivity of HSV-1 [96], which may due to potent strategies HSV-1 evolved to antagonize autophagy. In fact, HSV-1 ICP34.5 was reported to inhibit autophagy through a physical interaction with Beclin 1 and control the formation of autophagosomes in fibroblasts and neurons [97, 98].

### ER stress

A variety of cellular stresses can severely perturb endoplasmic reticulum (ER) function, also known as “ER stress”, triggering unfolded protein response (UPR) to preserving ER homeostasis. There are currently three UPR signaling pathways, distinguished by the action of three ER-localized transmembrane proteins inositol-requiring protein-1α (IRE1α), protein kinase RNA (PKR)-like ER kinase (PERK), and activating transcription factor 6 (ATF6) [15, 99]. HSV-1 glycoprotein gB were reported to selectively inhibit PERK activation [100]. Burnett et al. found that ICP0 promoter was responsive to ER stress which indicated that ICP0 might act as a sensor of cellular ER stress response to efficiently inhibit the UPR early in viral infection [101].

### SGs

Cellular stresses as well as viral infection could induce the formation of cytoplasmic proteins and RNA aggregates also known as stress granules (SGs). Nucleoprotein aggregates in SGs usually result in further storage, degradation in another granular compartment, or restart of translation upon recovery from stress. SGs are considered to be antiviral as increasing evidence show that SG formation and viral propagation are negatively correlated in multiple viral replication systems [102–104]. Finnen et al. found that HSV-2 infection did not cause accumulation of SGs and identified HSV-2 vhs as a virion component required for the disruption of SG formation [105]. In addition, HSV-1 protein ICP8 was found to inhibit SG formation by binding to G3BP [106], a crucial regulators of the formation and stability of SGs.

### Cell viability

Necrosis, a possible form of programmed cell death is a crucial strategy to control viral replication and pathogenesis [107, 108]. Treatment with tumor necrosis factor (TNF)-α triggers necrosis and results in the interaction between receptor-interacting kinase 1 (RIP1) and RIP3 through the RIP homotypic interaction motif (RHIM) domains [109]. Recent investigations have shown that HSV-1 R1 proteins ICP6 could prevent TNF-induced necroptosis in human cells. ICP6 could form complexes with both RIP1 and RIP3 via its RHIM domain hence abrogating the interaction between RIP1 and RIP3 [110, 111].

## Conclusion

**Fig. 1:**
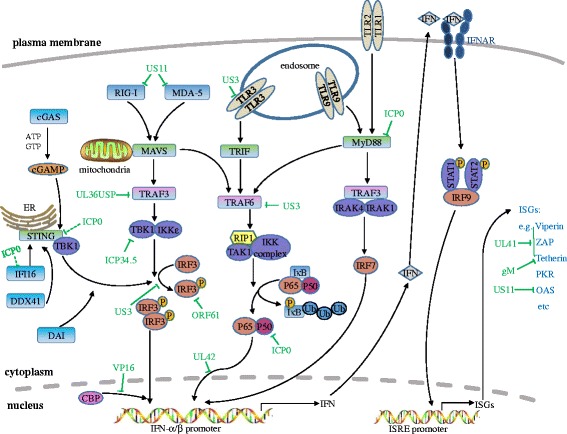
HSV-1 mediated evasion of the type I IFN signal pathway. PRRs, such as TLRs, RLRs and cytosolic DNA sensors, could recognize pathogen-associated molecular patterns. TLRs locate both at the plasma membrane and endosomes, and signal through TRIF and MyD88 to lead the activation of IRFs and NF-κB. RIG-I and MDA5 detect distinct RNA structures and signal through the adaptor protein MAVS to trigger IRF3 and NF-κB activation. cGAS recognizes dsDNA in the cytosol and subsequently catalyzes the production of cGAMP, a second messenger that activates the ER-localized adaptor protein STING. STING recruits and activates TBK1, which then activates IRF3 to induce type I IFNs. Multiple steps in the type I IFN signal pathway can be hijacked by HSV-1 proteins. Green full lines indicate confirmed interactions between host molecules and HSV-1 proteins. Green dashed lines indicate uncertain interactions that need to be further studied

In summary, the interplay between HSV-1 and the host antiviral innate immunity is very complicated, with viral components interference at multiple steps of the host antiviral defense signaling pathway (Fig. 1). Although the host has evolved multiple mechanisms to detect and trigger immediate antiviral responses, HSV-1 has also evolved strategies to counteract the host antiviral immune response. Notably, great progress has been made in the past few years in identifying novel host antiviral components, for example, the various cytosolic DNA sensors, it also brings the challenge for us to unravel the impact of these new elements on HSV-1 infection and the strategy HSV-1 evolved to cope with these effectors.
